# Ultra‐Processed Foods Reduction Enhances Clinical Outcomes and Dietary Profiles in Patients With Gingivitis: Results From a Randomised Controlled Trial

**DOI:** 10.1111/jcpe.70034

**Published:** 2025-09-14

**Authors:** Nicola Discepoli, Isabella De Rubertis, Giulia Tavella, Arianna Guazzelli, Styliani Konstantinidou, Barbara Paolini

**Affiliations:** ^1^ Unit of Periodontics, Department of Medical Biotechnologies University of Siena Siena Italy; ^2^ Unit of Dietetics and Clinical Nutrition, Department of Medical Sciences, Santa Maria Alle Scotte Hospital University of Siena Siena Italy

**Keywords:** gingivitis, Mediterranean diet, ultra‐processed food diet

## Abstract

**Aim:**

To evaluate the impact of ultra‐processed foods reduction advice (UPF‐RA) on gingivitis treatment and dietary patterns.

**Methods:**

Young adults with gingivitis were randomly assigned to two groups. At baseline, test group participants received UPF‐RA. At 8 weeks, professional mechanical plaque removal (PMPR) was carried out, followed by UPF‐RA (test group only). Full‐mouth periodontal charting and dietary data, collected through the NOVA Food Frequency Questionnaire and the Medi‐Lite, were recorded at baseline and at 8 and 16 weeks.

**Results:**

Sixty‐six patients (mean age: 23.3 ± 2.3 years; 32 males and 34 females) were included. At 8 weeks, a significant reduction in full‐mouth bleeding score (FMBS) was observed in the test group (18.9% ± 8.6% to 14.6% ± 9.0%; *p* = 0.04), with a concomitant decrease in UPF intake (912.7 ± 511.3 kcal to 446.9 ± 264.6 kcal; *p* < 0.001). No significant changes in FMBS and UPF consumption were observed in the control group (19.8% ± 9.0% to 19.1% ± 8.6%, *p* = 0.93; 776.4 ± 453.6 kcal to 775.3 ± 451.03, *p* = 1.00, respectively). At 16 weeks, gingivitis was resolved in 24% more cases in the test group. Logistic regression identified low UPF intake and UPF‐RA as significant predictors of FMBS reduction.

**Conclusions:**

Reduction in UPF consumption improved gingivitis treatment outcomes and participants' dietary quality. Patients with higher UPF consumption showed higher bleeding scores.

## 1 Introduction

Plaque‐induced gingivitis is an inflammatory response of the gingival tissues elicited by the accumulation of microbial biofilm, the management of which represents a primary prevention strategy for periodontitis (Chapple et al. 2018; Murakami et al. 2018; Tonetti et al. 2015). Gingivitis affects individuals of all ages and genders, exhibiting a high global prevalence and representing the most common manifestation of periodontal disease (Dye 2012; Trombelli et al. 2018; White et al. 2012). The onset of gingivitis occurs when the symbiotic relationship between the biofilm and the host's immune‐inflammatory response is disrupted (dysbiosis). Local and systemic modifying factors modulate the magnitude and progression of the immune‐inflammatory response; and among these, nutritional factors play a significant role (Chapple et al. 2018).

Ultra‐processed food (UPF) consumption (Marino et al. 2021), a cornerstone of the Western diet, has increased substantially, with evidence documenting an inverse relationship between UPF consumption and Mediterranean diet adherence (Bonaccio et al. 2021; da Rocha et al. 2021; Dinu et al. 2022). The NOVA classification categorises foods by industrial processing level into four groups: unprocessed or minimally processed foods (MPFs), processed culinary ingredients (PCIs), processed foods (PFs) and ultra‐processed foods (UPFs) (Monteiro et al. 2018; Monteiro et al. 2019). UPFs are energy‐dense but poor in proteins, dietary fibres, essential micronutrients and bioactive compounds (Fardet 2016; Scott 2017; Fardet et al. 2018). Their elevated content of rewarding ingredients (e.g., refined sugars) can lead to dopaminergic sensitisation and withdrawal symptoms (Schulte et al. 2015; LaFata et al. 2024), determining addiction‐like behaviour comparable to tobacco products (Gearhardt and DiFeliceantonio 2023). UPFs have been linked to oxidative stress, gut microbiota alterations and systemic low‐grade inflammation (Tristan Asensi et al. 2023). High consumption of UPFs is associated with increased risk of cerebrovascular disease, obesity and metabolic syndrome (Pagliai et al. 2021). A linear dose–response relationship exists between high UPF consumption and increased all‐cause mortality (Bonaccio et al. 2021). Pro‐inflammatory dietary patterns exacerbate gingival inflammatory response to biofilm accumulation and are associated with higher periodontitis risk (Alves‐Costa et al. 2024; Koelman et al. 2022; Machado et al. 2021; Weon Choi et al. 2023; Woelber, Gebhardt, and Hujoel 2023). Recent studies focusing on nutritional interventions in reducing gingival inflammation have demonstrated positive short‐term results (Bartha et al. 2022; Eberhard et al. 2022; Mainas et al. 2025; Woelber et al. 2019; Woelber, Reichenbächer, et al. 2023; Pappe et al. 2025).

Therefore, in this study we aimed to evaluate the effect of UPF reduction advice (UPF‐RA) on clinical outcomes of gingivitis treatment and on modulation of patients' dietary profiles.

## 2 Materials and Methods

### 2.1 Study Design

**FIGURE 1:**
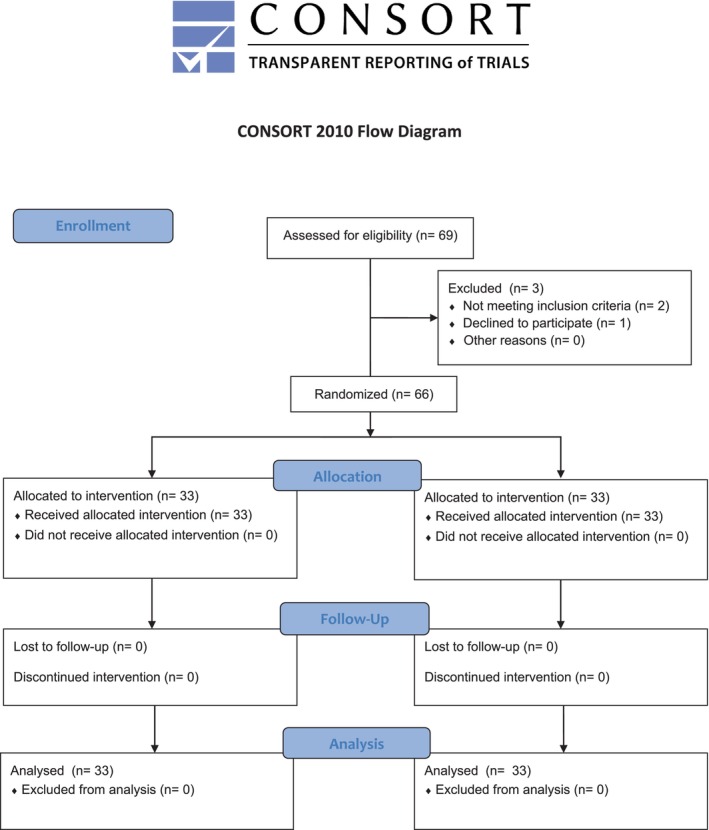
CONSORT diagram of participant flow through the trial.

The present randomised, parallel‐arm, single‐blind, University‐based, superiority clinical trial followed the CONSORT statement for clinical trials (Figure 1) and was conducted following the principles of the Declaration of Helsinki. The study protocol was approved by the University Hospital of Siena Ethics committee (Sezione Area vasta Toscana Sud‐Est, no. 26371) and registered on Clinicaltrials.gov (NCT06411535). Participants were informed about the protocol, and they provided written informed consent.

### 2.2 Setting and Participants

Participants were consecutively recruited among students enrolled in the medicine, dentistry and dental prosthodontics, and dental yygiene degree programmes at the University of Siena and screened for eligibility. All procedures were conducted at the Unit of Periodontics, Department of Medical Biotechnologies, AOUS, Le Scotte University Hospital (Siena), between May and October 2024. Participants meeting the following inclusion criteria were enrolled: (i) age between 18 and 40 years; (ii) absence of CAL loss due to periodontitis; (iii) absence of clinical signs suggestive of radiographic bone loss (radiographs were taken only when justified by clinical suspicion or by the presence of local factors that could account for attachment loss); (iv) bleeding on probing (BOP) ≥ 10%; and (v) ability to give written informed consent. Exclusion criteria were (i) orthodontic therapy or occlusal splints; (ii) systemic disease/conditions influencing gingival inflammation or dietary behaviour (e.g., pregnancy, diabetes, neutrophil defects, immune disorders); (iii) medications interfering with gingival response (female hormone replacement therapies, anti‐inflammatory agents, diphenylhydantoin, calcium channel blockers, cyclosporin A, immunostimulants/immunomodulators); and (iv) subgingival caries and/or inadequate restorations.

### 2.3 Phase 1

**FIGURE 2:**
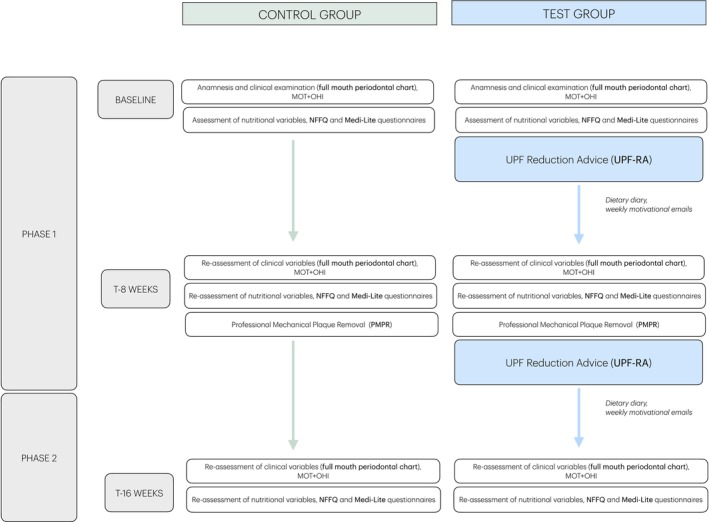
Timeline of clinical and dietary interventions across study phases.

The study chronogram is detailed in Figure 2.

#### 2.3.1 Baseline (T0) Anamnesis and Clinical Examination

Patients' demographic information, presence of family history of periodontitis as well as food allergies and/or intolerances were recorded. At T0, all enrolled participants underwent a full‐mouth periodontal charting. Examiners' training and blinding are detailed in Appendix 1. The following clinical parameters were recorded at six sites per tooth, with a periodontal probe (UNC 15, HuFriedy, Chicago, IL, USA) and a probing pressure of 0.25 N: probing pocket depth (PPD), gingival recession (REC), plaque control record (O'Leary et al. 1972) and bleeding on probing (BOP) (Ainamo and Bay 1975). CAL was calculated as the sum of PPD and REC. Full‐mouth plaque score (FMPS) and full‐mouth bleeding score (FMBS) were calculated as the proportion of all tooth surfaces that exhibited plaque and bleeding, respectively, upon probing. A diagnosis of gingivitis, either localised or generalised, was made in accordance with the EFP/AAP classification (Chapple et al. 2018; Trombelli et al. 2018).

Following baseline assessment, patients attended a session on oral hygiene instructions (OHIs) and motivation (Sanz et al. 2020).

#### 2.3.2 NOVA Food Frequency Questionnaire, Medi‐Lite Score, Oral Health Impact Profile 14

Following the clinical examination, patients were provided with (i) a questionnaire on the quality of food intake (NOVA food frequency questionnaire [NFFQ]; Dinu et al. 2021), (ii) a questionnaire on adherence to the Mediterranean diet (Medi‐Lite score; Sofi et al. 2017) and (iii) a questionnaire on the impact of oral health on quality of life (OHIP‐14; Slade 1997; Corridore et al. 2014). Further details are provided in Appendix 1.

#### 2.3.3 Ultra‐Processed Food Reduction Advice (UPF *‐* RA)

Patients in the test group alone additionally received UPF‐RA, delivered by a dentist (S.K.) who was not involved in the clinical examination and had previously been trained by a dietitian (G.T.) in nutritional science, behavioural strategies and communication techniques (details in Appendix 1). The training and calibration were coordinated by the Unit of Dietetics and Clinical Nutrition, Department of Medical Sciences, Santa Maria Alle Scotte Hospital, University of Siena.

The UPF‐RA was delivered chairside and included two phases: (a) an initial educational phase on UPFs and their health implications, and (b) a second phase aimed at identifying commonly consumed UPFs and advising on how to substitute them with unprocessed or MPF alternatives. A dietary diary and motivational materials were provided to support adherence at home (Appendix 1). The control group did not receive any dietary advice throughout the study.

#### 2.3.4 Phase 2 (T8)

Eight weeks after the baseline assessments, the full‐mouth periodontal charting was repeated, and tailored OHI was reinforced. Both groups attended a session on professional mechanical plaque removal (PMPR; Needleman et al. 2015), conducted by the same clinical operators (A.G., I.D.R.) using a magnetostrictive device (Cavitron Select SPS, Dentsply Sirona) and a rubber cup mounted on a low‐speed handpiece. Participants completed the questionnaires as part of the scheduled assessment. For patients in the test group, the UPF‐RA was repeated.

### 2.4 Phase 2 (T16)

The re‐evaluation visits were scheduled 16 weeks after baseline; clinical and dietary variables were reassessed during the same appointment.

#### 2.4.1 Outcome Measures and Sample Size Calculation

The primary outcome of the study was the reduction in BOP. The sample size calculation was based on the results of a previous randomised clinical trial (Graziani et al. 2018), which reported a mean BOP reduction of 6.66% (±18%) in the test group, which was receiving the nutritional intervention after 2 months of follow‐up. Student's *t*‐test was performed, assuming an alpha risk of 5% and a statistical power of 90% between paired observations. A required sample size of 60 individuals was obtained. To account for potential dropouts, estimated at 10%, the final experimental sample comprised 66 participants.

### 2.5 Randomisation, Blinding and Allocation Concealment

Randomisation and allocation concealment were ensured by an investigator who was not involved in the clinical trial. Patients were randomly assigned to the test or control group using a computer‐generated sequence (Stata IC18). Group allocation was concealed from clinical examiners throughout the study. Details are presented in Appendix 2.

### 2.6 Statistical Analysis

Statistical analysis was performed using an ad hoc software (Stata IC, version 18, Stata Corp LP, College Station, Texas). Continuous data were expressed as means and standard deviations (SDs), and binomial and categorical variables as counts and proportions. The Shapiro–Wilk test was used to test the normality of distribution of the data.

Comparisons between intervention groups were analysed with Student's *t*‐test. Intra‐group differences were computed with the repeated‐measures ANOVA and multiple comparisons using the Bonferroni correction.

Participants were classified based on their UPF consumption into low‐ and high‐UPF consumption groups, with the cut‐off set at the third quartile of UPF frequency intake distribution.

Multivariate logistic regression models were developed to assess the predictive role of patients' clinical characteristics, dietary patterns and UPF‐RA on bleeding reduction at 8 and 16 weeks. The Stata ‘allsets’ and ‘logistic’ commands were used for regression analysis. Changes (Δ) in FMBS from baseline to 8 and 16 weeks were dichotomised based on the median FMBS reduction and introduced as dichotomic dependent variables of the models.

## 3 Results

Sixty‐six patients (33 in the control group and 33 in the test group) were included at baseline. All patients received the allocated intervention and attended the scheduled follow‐up visits without any dropouts (Figure 1).

### 3.1 Phase 1

#### 3.1.1 (T0) Demographic and Clinical Variables

**FIGURE 3:**
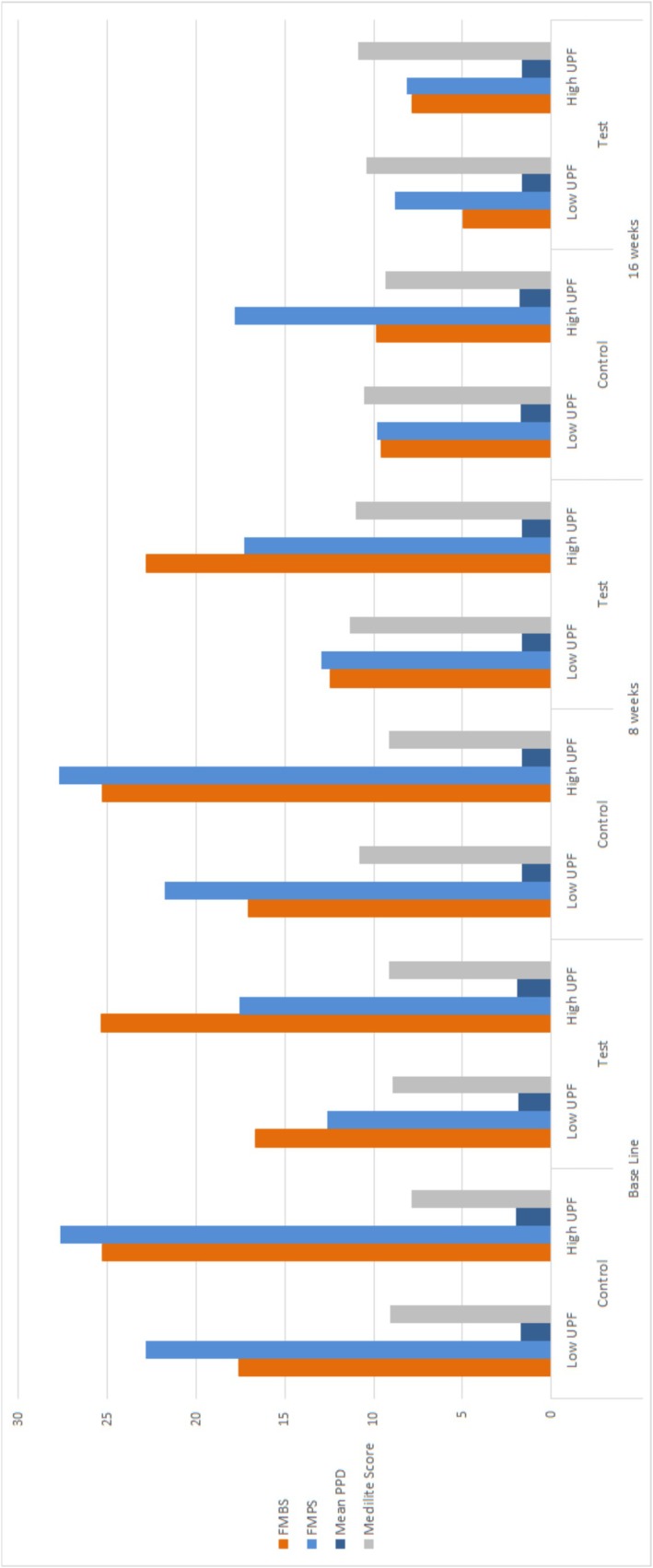
Subgroup analysis of clinical variables and Medi‐Lite score according to baseline UPF consumption.

Patient characteristics were homogeneously distributed between test and control groups (Table 1). Localised gingivitis was observed in 44 participants (66.7%), whereas generalised gingivitis was present in 22 participants (33.3%). Figure 3 shows the subgroup analysis of clinical variables according to UPF intake frequency. High UPF consumption (weekly UPF intake > 43.37 servings/week) was associated with significantly higher FMBS levels compared to low UPF consumers (weekly UPF intake ≤ 43.37 servings/week) in both groups (details in Tables [Link], [Link] and S3).

**TABLE 1:** Baseline demographic variables.

Variables		Control (*n* = 33)	Test (*n* = 33)
Gender (*N* [%])	Males	18 (54.55)	14 (42.41)
	Females	15 (45.45)	19 (57.59)
Age (Mean [SD])		23.51 (2.53)	23.15 (22.05)
BMI (kg/m^2^) (Mean [SD])		23.05 (2.58)	22.23 (2.85)
Family history of periodontitis (*N* [%])	No	29 (87.88)	31 (93.94)
	Yes	4 (12.12)	2 (6.06)
Allergies/intolerances (*N* [%])	No	27 (81.82)	28 (84.85)
	Yes	6 (18.18)	5 (15.15)
Type of allergy/intolerance (*N* [%])	Dried fruit	1 (16.67)	/
	Crustaceans	2 (33.33)	1 (20.00)
	Sulphites	/	1 (20.00)
	Lactose	/	2 (40.00)
	Strawberries	1 (16.67)	1 (20.00)
	Gluten	1 (16.67)	/
	Coconut	1 (16.67)	/
Toothbrush (*N* [%])	Manual	15 (45.54)	15 (45.54)
	Electric	18 (54.55)	18 (54.55)
Interproximal devices (*N* [%])	No	19 (57.58)	15 (45.45)
	Yes	14 (42.42)	18 (54.55)
Smoking status (*N* [%])	NS	21 (63.64)	26 (78.79)
	CS	11 (33.33)	7 (21.21)
	FS	1 (3.03)	/
Type of smoking (*N* [%])	EC	3 (27.27)	2 (28.57)
	TC	2 (18.18)	1 (14.29)
	HTP	6 (54.55)	4 (57.14)
Number of cigarettes per day (*N* [%])	< 10 per day	8 (72.73)	4 (57.14)
	≥ 10 per day	3 (27.27)	3 (42.86)
Physical activity (*N* [%])	No	2 (6.07)	9 (27.28)
	1–2 h/week	11 (33.33)	6 (18.18)
	3–4 h/week	8 (24.24)	8 (24.24)
	≥ 5 h/week	12 (36.36)	10 (30.30)

*Note*: < 10 per day, number and proportion of individuals smoking < 10 cigarettes per day; ≥ 10 per day, number and proportion of individuals smoking ≥ 10 cigarettes per day.

Abbreviations: BMI, body mass index; CS, current smoker; EC, electronic cigarette; FS, former smoker; HTP, heated tobacco product; NS, non‐smoker; TC, traditional cigarette.

#### 3.1.2 (T0) Nutritional Variables

Table 2 presents the consumption of MPF, PCI + PF and UPF. At baseline, no statistically significant differences were seen between the two groups (Table 2).

**TABLE 2:** Clinical and nutritional variables inter‐ and intra‐group comparisons.

Variables	Control (*n =* 33)			Test (*n =* 33)		
	Baseline	8 weeks	16 weeks	Baseline	8 weeks	16 weeks
%PPD < 4 mm (mean [SD])	97 (3.2)	97 (2.9)	98.5 (2.5)	96 (3.8)	99.2 (1.5)	99.4 (1.2)
%PPD ≥ 4 mm (mean [SD])	3 (3.2)	3 (2.9)	1.5 (2.5)	4 (3.8)	0.8 (1.5)	0.6 (1.2)
%PPD ≥ 5 mm (mean [SD])	1.4 (2.3)	1.3 (2.1)	0.6 (1.2)	0.8 (1.5)	0.2 (0.5)	0.2 (0.5)
Mean PPD (mean [SD])	1.77 (0.31)	1.70 (0.19)	1.69 (0.20)	1.89 (0.21)	1.67 (0.19)	1.65 (0.18)
FMBS (mean [SD])	19.78 (8.99)*	19.08 (8.60)*,**	9.78 (5.72)*,**	18.92 (8.59)*	14.64 (9.04)*,**	7.13 (4.58)*,**
FMPS (mean [SD])	24.04 (15.84)*,**	23.22 (19.05)*,**	11.76 (9.03)*	13.83 (10.51)**	11.99 (10.63)**	8.64 (6.05)
Mean REC (mean [SD])	0.1 (0.1)	0.1 (0.2)	0.1 (0.2)	0.1 (0.3)	0.1 (0.1)	0.1 (0.1)
Gingivitis extent *N* [%]						
Localised	24 (72.73)*	22 (73.33)	11 (68.75)*	20 (60.61)*	18 (75)	7 (87.50)*
Generalised	9 (27.27)	8 (26.67)	5 (31.25)**	13 (39.39)*	6 (25)*	1 (12.50)*,**
Unresolved gingivitis cases (*N* [%])	/	30 (90.91)	16 (48.48)**	/	24 (72.72)	8 (24.24)**
OHIP‐14 tot. (mean [SD])	6.88 (3.25)	5.42 (3.03)	5.82 (2.10)	7.09 (2.90)*	5.88 (2.70)	5.18 (2.00)*
MPF frequency intake (weekly) (mean [SD])	59.08 (19.07)	51.94 (17.65)	51.95 (17.62)	65.06 (20.37)	59.89 (19.39)	59.91 (19.39)
PCI + PF frequency intake (weekly) (mean [SD])	41.24 (12.89)	37.15 (13.25)	37.18 (13.35)	43.62 (18.37)	34.85 (13.53)	35.67 (13.59)
UPF frequency intake (weekly) (mean [SD])	38.5 (16.34)	33.03 (14.60)**	33.09 (14.51)**	35.5 (13.60)*	19.45 (9.52)*,**	19.94 (9.80)*,**
MPF energy share (daily, kcal) (mean [SD])	917.84 (431.53)	729.20 (271.68)**	714.41 (235.84)**	1133.96 (697.97)*,**	930.49 (452.42)**	918.18 (454.90)*
PCI + PF energy share (daily, kcal) (mean [SD])	950.37 (310.78)	793.084 (294.30)	798.79 (299.28)	1015.47 (581.62)	778.06 (481.58)	785.74 (461.94)
UPF energy share (daily, kcal) (mean [SD])	1002.68 (495.01)	776.45 (453.64)**	775.32 (451.07)**	912.74 (511.29)*	446.90 (264.59)*,**	437.69 (261.81)*,**
Total caloric intake (daily, kcal) (mean [SD])	2870.88 (890.25)*	2298.73 (674.79)*	2288.51 (672.43)*	3062.17 (1446.28)*	2155.73 (886.73)*	2124.60 (881.69)*
Medi‐Lite score (mean [SD])	8.79 (2.59)*	10.42 (2.41)*	10.27 (2.35)*	9 (2)*	11.24 (2.39)*	10.55 (1.99)*

Abbreviations: FMBS, full‐mouth bleeding score; FMPS, full‐mouth plaque score; MPF, minimally processed food; OHIP‐14 tot., total score of the Oral Health Impact Profile 14; PCI, processed culinary ingredient; PF, processed food; PPD, probing pocket depth; REC, recession; UPF, ultra‐processed food.

* *p*‐value < 0.05 for intra‐group comparisons.

** *p*‐value < 0.05 for inter‐group comparisons.

At T0, UPFs accounted for 34.1% and 30.7% of the total daily caloric intake in the control and test groups, respectively (Figure S1).

#### 3.1.3 (T8) Clinical Variables

A statistically significant reduction in FMBS was observed only in the test group between baseline and 8 weeks (18.92 ± 8.59 to 14.64 ± 9.04, *p* = 0.04). In the control group, FMBS showed no significant changes (19.78 ± 8.99 to 19.08 ± 8.60, *p* = 1.00). At 8 weeks, FMBS was significantly lower in the test group compared with the control group (inter‐group *p* = 0.04). No statistically significant intra‐group changes in FMPS were observed.

#### 3.1.4 (T8) Nutritional Variables

In the test group, the daily energy share from UPFs significantly decreased from baseline to week 8 (912.74 ± 511.29 to 446.90 ± 264.59 kcal, *p* < 0.001), and the Medi‐Lite score showed a statistically significant increase. At week 8, UPF intake was significantly higher in the control group (776.45 ± 453.64 kcal, *p* < 0.001) (Table 2).

MPF's contribution to total daily energy share increased from 36.7% to 42.9%, with a concomitant UPF reduction (30.7% to 22.2%; Figure S1).

### 3.2 Phase 2

#### 3.2.1 (T16) Clinical Variables

After plaque removal, FMBS decreased significantly in both groups compared to baseline and T8 values. At 16 weeks, FMBS was 7.13 ± 4.58 and 9.78 ± 5.72 in the test and control group, respectively (inter‐group *p* = 0.03). Gingivitis was still present in 24% of patients in the test group and 48% in the control group (Table 2).

A statistically significant reduction in the OHIP‐14 total score was recorded in the test group (from 7.09 ± 2.90 to 5.18 ± 2.00, *p* = 0.04), derived mainly from the domains of Physical Pain (items 3 and 4), Psychological Discomfort (items 5 and 6) and Social Disability (items 9 and 10).

Figure 3 shows that, in both groups, patients with lower baseline UPF consumption exhibited lower FMBS values throughout the study period.

Subgroup analyses by smoking status (Table S4) did not reveal any statistically significant differences.

#### 3.2.2 (T16) Nutritional Variables

In the test group, UPF consumption significantly decreased from baseline to 16 weeks (from 912.74 ± 511.29 to 437.69 ± 261.81 kcal/day; *p* < 0.001) and remained lower than in the control group (775.32 ± 451.07 kcal/day; *p* < 0.001) (Table 2).

### 3.3 Regression Models

Table 3 presents the results of the logistic regression analysis, considering the change (Δ) in FMBS between baseline and 8 weeks. The model identified an association between lower baseline FMBS levels (OR = 5.91 [1.21–28.91], *p* = 0.03) and low baseline UPF intake (OR = 7.34 [1.00–53.68], *p* < 0.001) with higher odds of greater FMBS reduction (Table 3).

**TABLE 3:** Logistic regression analysis of the change in FMBS between baseline and 8 weeks.

ΔFMBS Baseline – 8 weeks (AUC = 0.74; AIC = 71.8; BIC = 82.7)						
LR Chi^2^	Prob > Chi^2^	Pseudo‐*R* ^2^				
10.73	0.03	0.15			95% CI	
	OR	SE	Z	*p*	Lower	Higher
UPF‐RA	3.26	2.23	1.73	0.08	0.86	12.49
Lower baseline FMBS	5.91	4.79	2.19	**0.03**	1.21	28.91
Smoking status						
Smoker	0.45	0.36	−0.99	0.32	0.09	2.14
Low baseline UPF frequency intake	7.34	7.45	−3.08	**0.00**	1.00	53.68
_cons	0.24	0.03	−0.99	0.002	0.002	0.26

*Note*: Lower baseline FMBS, full‐mouth bleeding score at baseline ≤ 16.7%; low‐baseline UPF frequency intake, UPF frequency intake at baseline ≤ 43.37. The bold relates to the statistical significance of the value (*p* < 0.05) in comparison to non‐significance.

Abbreviation: UPF‐RA, ultra‐processed foods reduction advice.

In the model assessing the Δ between baseline and 16 weeks (Table 4), low UPF intake remained a significant predictor of FMBS improvements (OR = 0.06 [0.01–0.28], *p* < 0.001). Additionally, UPF‐RA was significantly associated with higher odds of a greater FMBS reduction over 16 weeks (OR = 0.21 [0.04–0.97], *p* = 0.04).

**TABLE 4:** Logistic regression analysis of the change in FMBS between baseline and 16 weeks.

Δ FMBS Baseline – 16 weeks (AUC = 0.83; AIC = 61.0; BIC = 69.8)						
LR Chi^2^	Prob > Chi^2^	Pseudo‐*R* ^2^				
20.09	0.00	0.28			95% CI	
	OR	SE	Z	*p*	Lower	Upper
UPF‐RA	0.21	0.16	−2.00	**0.04**	0.04	0.97
Interdental devices	1.81	1.30	0.82	0.41	0.44	7.42
Low baseline UPF frequency intake	0.06	0.05	−3.63	**0.00**	0.01	0.28
_cons	2.82	2.18	1.35	0.18	0.62	12.78

*Note*: Interdental devices, daily use of interdental devices; low baseline UPF frequency intake, UPF frequency intake at baseline ≤ 43.37. The bold relates to the statistical significance of the value (*p* < 0.05) in comparison to non‐significance.

Abbreviation: UPF‐RA, ultra‐processed foods reduction advice.

## 4 Discussion

This study evaluated the modulatory clinical effect of UPF‐RA on both gingival inflammatory parameters in the treatment of gingivitis, patients' dietary patterns and overall diet quality.

The clinical performance of the group receiving repeated sessions of UPF‐RA proved superior to that of the control group, with a lower proportion of residual gingivitis cases at both 8 (unresolved gingivitis: 72% vs. 90%) and 16 weeks of follow‐up (unresolved gingivitis: 24% vs. 48%). At the end of phase 1 (week 8), UPF‐RA seemed to contribute to the improvement of both patients' gingival inflammatory status and quality of diet. During this phase, no PMPR was performed in either group, so the observed reduction is most likely due to the reduction in UPF intake rather than to plaque reduction. Additionally, following PMPR (phase 2), both control and test groups experienced significant reductions in bleeding and plaque indices compared to baseline and 8‐week values. At 16 weeks, FMBS was still significantly lower in the test group compared to the control group, supporting the benefit of the UPF‐RA. Peri et al. (2022) evaluated the effectiveness of gingivitis treatment and found that after 1 month of professional mechanical treatment alone, < 10% of patients achieved gingivitis resolution. In contrast, when weekly oral hygiene instructions were combined with professional supragingival scaling and polishing, the resolution rate increased to 90% (Peri et al. 2022). These findings highlight the limited impact of professional treatment in the absence of any behavioural support strategy. In our experiment, logistic regression analyses confirmed the relevant role of both initial dietary profiles (low baseline UPF consumption) and UPF‐RA in predicting improvements in FMBS, as supported by the observed associations.

At baseline, UPF consumption was similar between groups, with no significant inter‐group differences in either dietary patterns or Medi‐Lite scores. These values exceeded the average daily caloric intake from UPFs reported in the general Italian population (Godos et al. 2022). This discrepancy may be attributed to the demographic characteristics of the study sample, younger age, occupational factors and dynamic lifestyles (Bonaccio et al. 2021; Lane et al. 2023; Ruggiero et al. 2021). Such findings indicate that approximately one‐third of total energy intake in our cohort (students enrolled in Health Sciences programmes) was derived from UPFs. The recorded data are consistent with the proportion of daily UPF‐derived energy intake observed across European countries (da Rocha et al. 2021; Julia et al. 2018; Latasa et al. 2018; Vandevijvere et al. 2019). A survey conducted on a cohort of university students from different academic fields (social sciences, humanities, engineering and health sciences) identified high UPF intake and significant misperception of dietary quality within this population, with no differences across study areas (Fondevila‐Gascón et al. 2022). This may be due to both psychological and behavioural expressions of autonomy in students living independently for the first time, as well as to scarce nutritional education.

At 16 weeks of follow‐up, the energy share from UPFs decreased significantly in the test group, along with the total daily caloric intake, pointing to the impact of UPF‐RA on the overall dietary pattern. Additionally, the reduction in UPF intake was accompanied by a significant increase in MPF consumption and in Medi‐Lite scores. The latter was driven by increased consumption of vegetables and legumes, along with a reduction in cereals (e.g., packaged products such as biscuits) and processed meat intake. Consistently, findings from recent studies showed an inverse relationship between a UPF‐rich diet and adherence to MD (Bonaccio et al. 2021; da Rocha et al. 2021; Dinu et al. 2022). The decrease in UPF consumption in the control group, despite no UPF‐RA, may reflect a Hawthorne effect (McCarney et al. 2007) resulting from completing the NFFQ and MEDI‐LITE, which likely increased dietary awareness and prompted behavioural changes.

Moreover, our results demonstrate that patients with lower baseline UPF consumption consistently exhibited lower bleeding scores throughout the follow‐up period.

Previous studies have shown that specific dietary patterns (Hujoel 2009; Marruganti et al. 2022; Yue et al. 2024) and nutritional interventions are associated with better periodontal biometric indices (Graziani et al. 2018; Woelber et al. 2019; Bartha et al. 2022; Staufenbiel et al. 2023; Pappe et al. 2025). Woelber et al. examined an anti‐inflammatory diet (AID) that reduced processed carbohydrates and animal proteins while increasing micronutrients. They reported significant reduction in gingival index following the AID protocol without PMPR (Woelber et al. 2019). Similarly, Bartha and coworkers evaluated the effects of an MD intervention on gingival inflammatory parameters. They observed a significant reduction in BOP in the test group between baseline and 6 weeks of follow‐up (50.48 ± 12.2 vs. 39.93 ± 13.7, respectively; 21% relative reduction), demonstrating that the shift of patients' dietary pattern to MD had a beneficial effect on gingival inflammation (Bartha et al. 2022). Such clinical implications may be attributed to the local effects of food ingredients, such as fermentable carbohydrates, capable of inducing a pathogenetic shift in the commensal oral biofilm (Marsh 2003, 2006), with the establishment of a dysbiotic oral environment affecting host immune modulation (Demmer et al. 2017). In addition, the detrimental impact of unhealthy dietary habits on systemic health and immune regulation (Sofi et al. 2008) provides a rationale for the role of diet on gingival inflammatory parameters. An MD, low in UPF and rich in fibres, antioxidants and omega‐3 fatty acids, is associated with lower systemic inflammatory markers and reduced insulin resistance (Esposito et al. 2004), with positive effects on overall periodontal health (Demmer et al. 2017).

Dietary products typical of a UPF‐rich diet, such as refined sugars, salt, saturated fats and food processing substances (Monteiro et al. 2018; Monteiro et al. 2019; Moubarac et al. 2014), have been associated with chronic low‐grade inflammation (Christ et al. 2019; Thorburn et al. 2014; Tristan Asensi et al. 2023), a state that modulates immune response and exacerbates inflammatory conditions, including gingivitis. Specifically, UPF‐rich diets have been linked to postprandial hyperglycaemia, increased oxidative stress after meals and elevated pro‐inflammatory cytokine production, ultimately exacerbating periodontal immune response (Giugliano et al. 2006; Woelber and Vach 2023).

Although several studies have documented the association between the consumption of food ingredients falling into the UPF category (e.g., sugar, white flour, soft drinks) and increased odds of both periodontitis and gingivitis (Lula et al. 2014; Woelber, Reichenbächer, et al. 2023; Alves‐Costa et al. 2024), Bidinotto and coworkers, who investigated the association between UPF intake and periodontitis using NHANES data, found no significant relationship (Bidinotto et al. 2022). However, the periodontal classification in NHANES is based on variables that may not fully capture disease status, and the lack of detailed food items recording, along with the use of 24‐h dietary recalls, may have limited the assessment of actual dietary patterns.

Several limitations should be considered when interpreting these results. The study population consisted of young university students with high average UPF consumption. University students often exhibit unhealthy lifestyles (Castelao‐Naval et al. 2019), including high UPF consumption (Fondevila‐Gascón et al. 2022). This demographic specificity may limit the generalisability of our findings to older populations or different socioeconomic backgrounds. The cohort comprised students of medicine, dentistry and dental hygiene, who might have been particularly responsive to the topic. Their awareness of participating in a university clinical trial may have contributed to high compliance and absence of dropouts. Dietary data were self‐reported and therefore subject to recall or social desirability bias. UPF‐RA should not be compared with a full nutritional programme by a dietitian, but rather as a risk‐factor control strategy routinely introducible in dental settings. Future studies should assess the clinical impact of UPF reduction interventions in different populations and age groups and evaluate whether dietary improvements are maintained over time.

## 5 Conclusions

This study suggests that a UPF‐RA delivered within a dental setting can significantly improve gingival inflammatory parameters in young adults undergoing gingivitis treatment. It demonstrated a positive effect on overall dietary patterns and contributed to reduced daily caloric intake. These findings support integrating dietary assessment into periodontal care protocols, aligning with the growing emphasis on host‐modulating factors in periodontal disease management.

## Author Contributions

**Nicola Discepoli:** conceptualization, methodology, writing – original draft, formal analysis. **Isabella De Rubertis:** methodology, formal analysis, writing – original draft. **Giulia Tavella:** investigation, methodology. **Arianna Guazzelli:** investigation, writing – review and editing. **Styliani Konstantinidou:** investigation, writing – review and editing. **Barbara Paolini:** conceptualization, writing – review and editing, formal analysis.

## Consent

All enrolled patients were informed about the study protocol and were asked to read and sign the informed consent.

## Conflicts of Interest

The authors declare no conflicts of interest.

## Supporting information


**Table S1:** Distribution of clinical variables and Medi‐Lite score according to the UPF consumption, overall cohort.

**Table S2:** Distribution of clinical variables and Medi‐Lite score according to the UPF consumption, control group.

**Table S3:** Distribution of clinical variables and Medi‐Lite score according to the UPF consumption, test group.

**Table S4:** Subgroup analysis of clinical variables according to the smoking status.

**Figure S1:** Daily energy share from food categories at baseline and at 8 and 16 weeks.

## Data Availability

The data that support the findings of this study are available on request from the corresponding author. The data are not publicly available due to privacy or ethical restrictions.

## Appendix 1

### Examiner Characteristics

Two masked and calibrated examiners (A.G., I.D.R.) performed the clinical measurements, using a periodontal probe (UNC 15, HuFriedy, Chicago, IL, USA) and a probing pressure of 0.25 N, six sites per tooth (Lang et al. 1991). Prior to the study start, the examiners underwent a training session on two non‐study subjects diagnosed with gingivitis, assessing clinical attachment level (CAL) and bleeding on probing (BOP) (ICC for continuous data, 0.92; unweighted Cohen's kappa for categorical data, 0.91).

Questionnaire characteristics:

### NOVA Food Frequency Questionnaire (NFFQ)

The NFFQ is a validated 94‐item questionnaire that enables the assessment of food consumption based on different levels of processing, following the NOVA classification system (Dinu et al. 2021; Monteiro et al. 2018), and resulting in three consumption categories: (i) unprocessed or minimally processed foods (MPFs), (ii) processed culinary ingredients and processed foods (PCIs + PFs) and (iii) ultra‐processed foods (UPFs) (Dinu et al. 2021). The 94 items are divided into nine domains: (i) Fruits and dried fruits; (ii) Vegetables and legumes; (iii) Cereals and tubers; (iv) Meat and fish; (v) Milk, dairy products and eggs; (vi) Oils, fats and condiments; (vii) Sweets and sweeteners; (viii) Beverages; (ix) Other (packaged sweet or savoury snacks, plant‐based beverages and yogurt, plant‐based substitutes for cheese and meat, powder drinks/shakes and other meal replacement products). Participants are asked to quantify the frequency of consumption of a given food item, choosing from the following options: ‘Never or less than once a month’; ‘1–3 times a month’; ‘Once a week’; ‘Twice a week’; ‘Three times a week’; ‘Four times a week’; ‘Five times a week’; ‘Six times a week’; ‘Every day’. Furthermore, they are asked to indicate the number of times per day every food is consumed. Finally, participants identify the portion size, choosing from 0.5, 1, 1.5, 2, 2.5 or 3. To help patients select the most accurate option, a reference portion size is provided next to each item.

Results from the NFFQ provided the weekly frequency of consumption for MPF, PCI + PF and UPF categories. The corresponding daily caloric intake for each food category was subsequently estimated, as described in section 2.3.2, by a dietitian (G.T.) using the Italian Food Composition Database from CREA and the BDA‐IEO Food Database, taking into account both portion sizes and consumption frequency.

### Medi‐Lite Score

The Medi‐Lite score is a validated tool designed to assess adherence to the Mediterranean diet based on literature‐derived dietary components (Sofi et al. 2017). The score evaluates dietary intake across nine food categories: (i) Fruits; (ii) Vegetables; (iii) Cereals; (iv) Legumes; (v) Fish and fish products; (vi) Meat and meat products; (vii) Dairy products; (viii) Alcohol intake; and (ix) Olive oil. For food groups characteristic of the Mediterranean diet (fruits, vegetables, cereals, legumes and fish), a score of 2 is assigned to the highest intake category, 1 to the middle category and 0 to the lowest category. Conversely, for food groups not traditionally associated with the Mediterranean diet (meat, meat products and dairy products), scoring is reversed, with 2 points assigned to the lowest intake category, 1 to the middle and 0 to the highest. Alcohol intake is scored based on daily consumption in alcohol units (1 unit = 12 g of alcohol), with 2 points assigned to moderate intake (1–2 units/day), 1 point for low intake (≤ 1 unit/day) and 0 points for excessive consumption. Additionally, 2 points are assigned for regular use of olive oil, 1 point for frequent use and 0 point for occasional use.

The final Medi‐Lite score was calculated as the sum of individual category scores, ranging from 0 (low adherence) to 18 (high adherence).

### Oral Health Impact Profile 14

Participants were then provided with the Oral Health Impact Profile 14 questionnaire (OHIP‐14), which aims to assess, from the patient's perspective, the impact of their oral health status on the quality of life. The OHIP‐14 encompasses 14 items and, for each question, participants can respond by choosing from the following scores on a Likert‐type scale from 0 to 4: 0 = never; 1 = hardly ever; 2 = occasionally; 3 = fairly often; 4 = very often (Slade 1997). Responses to single OHIP‐14 items can be summed up to obtain the questionnaire's total sum score, and the higher total score, the greaterthe impact on oral health–related quality of life. The validated Italian version was used (Corridore et al. 2014).

### Ultra‐Processed Food Reduction Advice (UPF‐RA)

The UPF‐RA was delivered chairside by a trained dentist (S.K.). The training session encompassed the principles of healthy dietary patterns, evidence‐based information on the systemic and oral health implications of ultra‐processed food (UPF) consumption and behavioural nutrition education approaches (Contento 2008) adapted specifically for dental practice settings. Emphasis was placed on patient‐centred communication and motivational strategies to optimise patient adherence to dietary modifications (Arnett et al. 2023; Bray et al. 2021; Crowe et al. 2023).

To guarantee accuracy and reproducibility in the delivery of the intervention, the dentist (S.K.) underwent two calibration sessions of Ultra‐Processed Food Reduction Counselling (UPF‐RC) delivered to two non‐study patients referred to the Unit of Dietetics and Clinical Nutrition, Department of Medical Sciences, Santa Maria Alle Scotte Hospital, University of Siena (Italy), under the supervision of the dietitian (G.T.). The UPF‐RC was delivered following a checklist to ensure accurate application of the key counselling components.

The UPF‐RA session for test group participants was structured to be both effectively delivered and appropriate to the time and context of a dental setting. It encompassed an initial educational phase, aimed at introducing patients to the concept of UPF, explaining what food processing involves and the health implications associated with their consumption.

The NOVA classification system was presented in detail. Additionally, an evidence‐based overview of the adverse effects of high UPF consumption on both systemic and oral health was provided.

The second part of the session aimed to teach patients how to recognise UPFs, particularly those frequently present in their own diet. To enhance compliance and motivation and to facilitate the recognition of the UPFs, participants received educational materials and a comprehensive list of commonly consumed UPFs. Participants were instructed to monitor and reduce their UPF consumption using a structured dietary diary. Practical advice was provided on how to replace UPFs with unprocessed and minimally processed alternatives. The diary was designed to allow participants to record their weekly consumption of foods across all processing categories (MPF, PCI, PF, UPF) and to track the dietary improvements. Additionally, participants in the test group received weekly motivational emails throughout the study duration to reinforce behavioural changes.

## Appendix 2

Randomisation and allocation concealment were guaranteed by an investigator who was not involved in the clinical trial. After enrolment, patients were randomly assigned to one of the two groups using a computer‐generated sequence (Stata IC18, command “rndseq”). Group allocation codes were put into sealed and opaque envelopes, with participants' identification numbers on the outside. The envelopes were opened by the clinician in charge of the dSNC (S.K.) only after assessment of the baseline demographic and clinical variables. The operators performing the clinical measurements remained blinded to group assignment throughout the study.
