# Supplementary material for: Ultra‐Processed Foods Reduction Enhances Clinical Outcomes and Dietary Profiles in Patients With Gingivitis: Results From a Randomised Controlled Trial
Source: J Clin Periodontol. 2025 Sep 14;53(1):12–25. doi: 10.1111/jcpe.70034 (PMC12695454; doi:10.1111/jcpe.70034)
Supplement: Supplementary file 1 — Table S1: Distribution of clinical variables and Medi‐Lite score according to the UPF consumption, overall cohort. [file JCPE-53-12-s005.docx]

**Supplementary Table 1** Distribution of clinical variables and Medi-Lite Score according to the UPF consumption, **overall cohort**

| Variables | Low UPF Frequency Intake | | | High UPF Frequency Intake | | |
| --- | --- | --- | --- | --- | --- | --- |
|  | **Baseline** | **8 weeks** | **16 weeks** | **Baseline** | **8 weeks** | **16 weeks** |
| FMBS  (Mean [SD]) | 17.25 (6.95)^§ ∆^ | 14.76 (7.39)^§∆^ | 7.26(5.98)^∆^ | 25.92 (10.59)^§∆^ | 24.04 (10.14)^§∆^ | 8.84(5.08)^∆^ |
| FMPS  (Mean [SD]) | 17.75 (13.44)^∆^ | 17.35 (15.11)^∆^ | 9.31 (6.83)^∆^ | 22.62 (16.64)^∆^ | 22.49 (19.72)^∆^ | 12.99 (9.98)^∆^ |
| Mean PPD  (Mean [SD]) | 1.79 (0.25) | 1.67(0 .19) | 1.67 (0.20) | 1.96 (0.30) | 1.73 (0.18) | 1.66 (0.18) |
| Medi-Lite Score  (Mean [SD]) | 9.02 (2.44)^∆^ | 11.08 (2.53)^∆^ | 10.5 (2.29)^∆^ | 8.5 (1.83)^∆^ | 10.06 (1.88) | 10.12 (1.71)^∆^ |

Abbreviations: FMBS, Full Mouth Bleeding Score; FMPS, Full Mouth Plaque Score; Mean PPD, average probing pocket depth; OHIP-14 tot, total score of the Oral Health Impact Profile 14.

^∆^ p-Value <0.05 for intra-group comparisons

^§^ p-Value <0.05 for inter-group comparisons
