# Supplementary material for: Ultra‐Processed Foods Reduction Enhances Clinical Outcomes and Dietary Profiles in Patients With Gingivitis: Results From a Randomised Controlled Trial
Source: J Clin Periodontol. 2025 Sep 14;53(1):12–25. doi: 10.1111/jcpe.70034 (PMC12695454; doi:10.1111/jcpe.70034)
Supplement: Supplementary file 2 — Table S2: Distribution of clinical variables and Medi‐Lite score according to the UPF consumption, control group. [file JCPE-53-12-s002.docx]

**Supplementary Table 2** Distribution of clinical variables and Medi-Lite Score according to the UPF consumption, **control group**

| Variables | Low UPF Frequency Intake | | | High UPF Frequency Intake | | |
| --- | --- | --- | --- | --- | --- | --- |
|  | **Baseline** | **8 weeks** | **16 weeks** | **Baseline** | **8 weeks** | **16 weeks** |
| FMBS  (Mean [SD]) | 17.63 (7.47)^§∆^ | 17.09 (7.76)^§ ∆^ | 9.58 (7.15)^∆^ | 25.29 (8.57)^§∆^ | 25.29 (8.57)^§∆^ | 9.85 (5.35)^∆^ |
| FMPS  (Mean [SD]) | 22.87 (15.21)^∆^ | 21.79 (18.36)^∆^ | 9.80 (7.41)^§∆^ | 27.67 (18.26) | 27.71 (21.71) | 17.86 (11.33)^§^ |
| Mean PPD  (Mean [SD]) | 1.71 (0.28) | 1.67 (0.19) | 1.71 (0.22) | 2.00 (0.32) | 1.64 (0.12) | 1.79 (0.19) |
| Medi-Lite Score  (Mean [SD]) | 9.08 (2.78) | 10.84 (2.47) | 10.56 (2.50) | 7.87 (1.72) ^∆^ | 9.13 (1.72) | 9.37 (1.59) |

Abbreviations: FMBS, Full Mouth Bleeding Score; FMPS, Full Mouth Plaque Score; Mean PPD, average probing pocket depth; OHIP-14 tot, total score of the Oral Health Impact Profile 14.

^∆^ p-Value <0.05 for intra-group comparisons

^§^ p-Value <0.05 for inter-group comparisons
