# Supplementary material for: Ultra‐Processed Foods Reduction Enhances Clinical Outcomes and Dietary Profiles in Patients With Gingivitis: Results From a Randomised Controlled Trial
Source: J Clin Periodontol. 2025 Sep 14;53(1):12–25. doi: 10.1111/jcpe.70034 (PMC12695454; doi:10.1111/jcpe.70034)
Supplement: Supplementary file 3 — Table S3: Distribution of clinical variables and Medi‐Lite score according to the UPF consumption, test group. [file JCPE-53-12-s004.docx]

**Supplementary Table 3:** Distribution of clinical variables and Medi-Lite Score according to the UPF consumption, **test group**

| Variables | Low UPF Frequency Intake | | | High UPF Frequency Intake | | |
| --- | --- | --- | --- | --- | --- | --- |
|  | **Baseline** | **8 weeks** | **16 weeks** | **Baseline** | **8 weeks** | **16 weeks** |
| FMBS  (Mean [SD]) | 16.86 (6.51)^§∆^ | 12.42 (6.32)^§ ∆^ | 4.94 (3.64)^∆^ | 25.33 (11.41)^§∆^ | 22.79 (11.97)^§∆^ | 7.82 (4.69)^∆^ |
| FMPS  (Mean [SD]) | 12.62 (9.08) | 12.93 (9.38) | 8.80 (6.30) | 17.58 (14.20) | 17.29 (17.32) | 8.12 (5.53) |
| Mean PPD  (Mean [SD]) | 1.88 (0.18) | 1.67 (0.20) | 1.64 (0.17) | 1.93 (0.29) | 1.68 (0.17) | 1.68 (0.23) |
| Medi-Lite Score  (Mean [SD]) | 8.96 (2.09) ^∆^ | 11.32 (2.61)^∆^ | 10.44 (2.12) | 9.12(1.81) | 11 (1.60) | 10.87 (1.55) |

Abbreviations: FMBS, Full Mouth Bleeding Score; FMPS, Full Mouth Plaque Score; Mean PPD, average probing pocket depth; OHIP-14 tot, total score of the Oral Health Impact Profile 14.

^∆^ p-Value <0.05 for intra-group comparisons

^§^ p-Value <0.05 for inter-group comparisons
