# Supplementary material for: Ultra‐Processed Foods Reduction Enhances Clinical Outcomes and Dietary Profiles in Patients With Gingivitis: Results From a Randomised Controlled Trial
Source: J Clin Periodontol. 2025 Sep 14;53(1):12–25. doi: 10.1111/jcpe.70034 (PMC12695454; doi:10.1111/jcpe.70034)
Supplement: Supplementary file 4 — Table S4: Subgroup analysis of clinical variables according to the smoking status. [file JCPE-53-12-s003.docx]

**Supplementary Table 4** Subgroup analysis of clinical variables according to the smoking status

| Variables | Control group (n=33) | | | | | |
| --- | --- | --- | --- | --- | --- | --- |
|  | **Current Smokers (*n=11*)** | | | **Non-Smokers/Former Smokers (*n=22*)** | | |
|  | **Baseline** | **8 weeks** | **16 weeks** | **Baseline** | **8 weeks** | **16 weeks** |
| Mean PPD  (Mean [SD]) | 1.61 (0.30) | 1.73 (0.17) | 1.66 (0.14) | 1.85 (0.29) | 1.69 (0.20) | 1.71 (0.23) |
| FMBS  (Mean [SD]) | 20.30 (10.68) | 21.19 (10.13) | 8.41 (4.77) | 19.52 (8.28) | 18.03 (7.77) | 10.45 (6.08) |
| FMPS  (Mean [SD]) | 26.19 (16.28) | 27.74 (20.33) | 14.53 (8.16) | 23.05 (15.95) | 20.94 (18.90) | 10.50 (9.51) |
| Mean REC  (Mean [SD]) | 0.1 (0.1) | 0.1 (0.2) | 0.1 (0.3) | 0.1 (0.2) | 0.1 (0.1) | 0.1 (0.1) |
| Gingivitis Extent  N [%]) |  |  |  |  |  |  |
| Localized | 8 (72.73) | 7 (63.64) | 4 (36.36) | 16 (72.73) | 12 (54.55) | 7 (31.82) |
| Generalized | 3 (27.27) | 4 (36.36) | 2 (18.18) | 6 (27.27) | 7 (31.82) | 3 (13.64) |
| Unresolved Gingivitis Cases  (N [%]) | / | 11 (100) | 6 (54.55) | / | 19 (86.36) | 10 (45.45) |
| Variables | **Test group (n=33)** | | | | | |
|  | **Current Smokers (*n=7*)** | | | **Non-Smokers (*n=26*)** | | |
|  | **Baseline** | **8 weeks** | **16 weeks** | **Baseline** | **8 weeks** | **16 weeks** |
| Mean PPD  (Mean [SD]) | 1.84 (0.19) | 1.72 (0.20) | 1.63 (0.18) | 1.91 (0.22) | 1.66 (0.19) | 1.65 (0.18) |
| FMBS  (Mean [SD]) | 17. 90 (8.49) | 15.36 (6.60) | 6.59 (3.36) | 19.19 (8.77) | 14.59 (9.67) | 7.27 (4.90) |
| FMPS  (Mean [SD]) | 20.58 (9.59) | 11.40 (8.47) | 7.95 (4.26) | 12 (10.16) | 12. 17 (10.71) | 8.83 (6.51) |
| Mean REC  (Mean [SD]) | 0.1 (0.1) | 0.1 (0.2) | 0.1 (0.3) | 0.1 (0.2) | 0.1 (0.1) | 0.1 (0.1) |
| Gingivitis Extent  N [%]) |  |  |  |  |  |  |
| Localized | 4 (57.14) | 4 (57.14) | 1 (14.29) | 16 (61.54) | 14 (53.85) | 5 (19.23) |
| Generalized | 3 (42.86) | 2 (28.57) | / | 10 (38.46) | 4 (19.23) | 2 (7.69) |
| Unresolved Gingivitis Cases  (N [%]) | / | 6 (85.71) | 1 (14.29) | / | 18 (69.23) | 7 (26.92) |

**Abbreviations:** PPD probing pocket depth; FMBS, Full Mouth Bleeding Score; FMPS, Full Mouth Plaque Score; REC, recession.
