# Supplementary figures and images for: Ultra‐Processed Foods Reduction Enhances Clinical Outcomes and Dietary Profiles in Patients With Gingivitis: Results From a Randomised Controlled Trial
Source: J Clin Periodontol. 2025 Sep 14;53(1):12–25. doi: 10.1111/jcpe.70034 (PMC12695454; doi:10.1111/jcpe.70034)

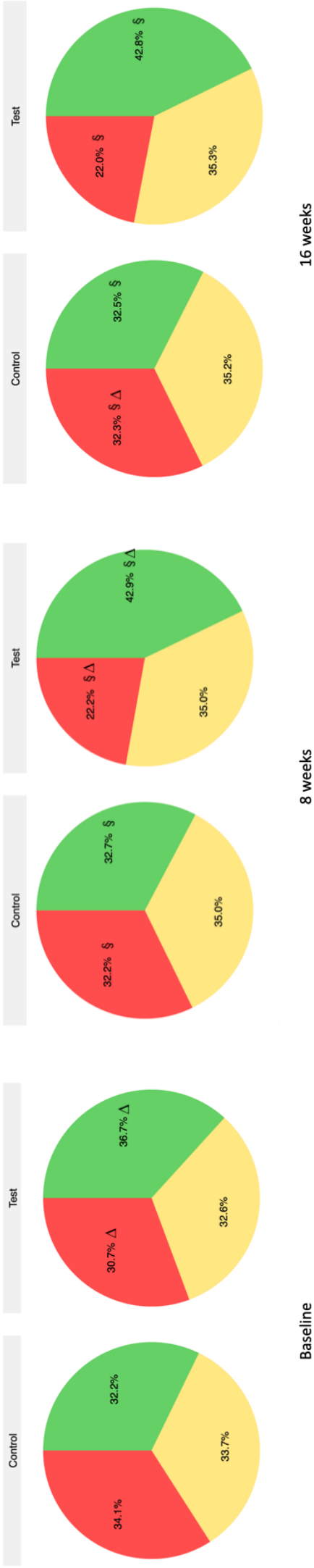

■ MPF  
■ PCI+PF  
■ UPF

$\Delta$  p-Value <0.05 for intra-group comparisons  
 $\S$  p-Value <0.05 for inter-group comparisons

Supplement: Supplementary file 5 — Figure S1: Daily energy share from food categories at baseline and at 8 and 16 weeks. [file JCPE-53-12-s001.pdf]
